# Effects of a Ketogenic Diet on Body Composition in Healthy, Young, Normal-Weight Women: A Randomized Controlled Feeding Trial

**DOI:** 10.3390/nu16132030

**Published:** 2024-06-26

**Authors:** Jonas Burén, Michael Svensson, Per Liv, Anna Sjödin

**Affiliations:** 1 Department of Food, Nutrition and Culinary Science, Umeå University, 90187 Umeå, Sweden; anna.sjodin@umu.se; 2 Umeå School of Sport Sciences, Umeå University, 90187 Umeå, Sweden; michael.svensson@umu.se; 3 Department of Community Medicine and Rehabilitation, Section of Sports Medicine, Umeå University, 90187 Umeå, Sweden; 4 Department of Public Health and Clinical Medicine, Section of Sustainable Health, Umeå University, 90187 Umeå, Sweden; per.liv@umu.se

**Keywords:** weight reduction, fat-free mass (FFM), DEXA, low-carbohydrate diet (LCD), high-fat diet (HFD), saturated fatty acids, carbohydrate restriction, metabolism, female, sports nutrition

## Abstract

This study investigates the effects of a ketogenic low-carbohydrate high-fat (LCHF) diet on body composition in healthy, young, normal-weight women. With the increasing interest in ketogenic diets for their various health benefits, this research aims to understand their impact on body composition, focusing on women who are often underrepresented in such studies. Conducting a randomized controlled feeding trial with a crossover design, this study compares a ketogenic LCHF diet to a Swedish National Food Agency (NFA)-recommended control diet over four weeks. Seventeen healthy, young, normal-weight women adhered strictly to the provided diets, with ketosis confirmed through blood β-hydroxybutyrate concentrations. Dual-energy X-ray absorptiometry (DXA) was utilized for precise body composition measurements. To avoid bias, all statistical analyses were performed blind. The findings reveal that the ketogenic LCHF diet led to a significant reduction in both lean mass (−1.45 kg 95% CI: [−1.90;−1.00]; *p* < 0.001) and fat mass (−0.66 kg 95% CI: [−1.00;−0.32]; *p* < 0.001) compared to the control diet, despite similar energy intake and physical activity levels. This study concludes that while the ketogenic LCHF diet is effective for weight loss, it disproportionately reduces lean mass over fat mass, suggesting the need for concurrent strength training to mitigate muscle loss in women following this diet.

## 1. Introduction

There has been a profound increase in interest in ketogenic diets over the past decade, attracting attention from both researchers and the general public. Initially seen as a method to combat obesity and diabetes, the therapeutic effects of ketogenic diets are now recognized across a broad range of conditions, including epilepsy, cancer, inflammation, and neurodegenerative diseases [1]. In addition, one of these new research areas concerns whether a ketogenic diet can improve body composition and/or physical performance among athletes as well as everyday exercisers. In a recently published systematic review, Kang et al. [2] examined the potential ergogenic effects of a ketogenic diet on healthy normal-weight individuals who adhered to a ketogenic diet for a minimum of three weeks. According to the 14 studies that reported on body composition, it seems that fat-free body mass (i.e., muscle) can be preserved, while fat mass decreases. However, as the authors of the systematic review highlighted, the majority (~85%) of participants in these studies were men. This gender disparity in research on the effect of diets on body composition is furthermore emphasized in the International Society of Sports Nutrition (ISSN) position stand [3]. It is a well-known fact that there are some biological differences between men and women. Sex chromosomes and sex-specific hormones, including androgens in males and estrogens and progesterone in females, affect energy metabolism [4]. For example, there appears to be a difference in substrate utilization when adapting to a ketogenic diet. A study of CrossFit-trained athletes showed that males appear to shift towards greater fat utilization during submaximal-intensity exercises under a ketogenic diet compared to female athletes [5]. Consequently, one cannot extrapolate data from men to women. The majority of the studies in Kang’s systematic review did not include women at all, and there was only one study [6] where the number of participating women was more than five individuals. However, the data were not broken down by sex in that study. Another recent systematic review by Murphy et al. [7] on ketogenic diets (≥two weeks) and performance in non-obese healthy individuals with more objectively defined ketosis (carbohydrate intake ˂ 50 g/day or circulating β-hydroxybutyrate ≥ 0.5 mM) found nine studies that reported on body composition. However, only a handful of these studies included women (with no more than ten women in any single study), and the authors suggested that sex may be one of several factors that contribute to discrepancies in the outcomes between studies. In addition, the authors call for future studies where food intake is carefully controlled in order to minimize differences in body mass change and thereby eliminate a potential confounding variable. 

In summary, there is a lack of body composition data from well-controlled and randomized feeding studies on young, healthy, normal-weight women who eat a truly ketogenic diet. In light of this, an explorative, randomized, and controlled feeding trial with a crossover design was conducted on young, healthy, normal-weight women following a non-energy-restricted ketogenic low-carbohydrate high-fat (LCHF) diet. A Swedish National Food Agency (NFA)-recommended diet was used as a control diet. The primary outcome was LDL cholesterol [8]. Here, we report the effects of a 4-week non-energy-restricted ketogenic LCHF diet on body composition in young, healthy, normal-weight women.

## 2. Materials and Methods

### 2.1. Study and Design Overview

**Figure 1:**
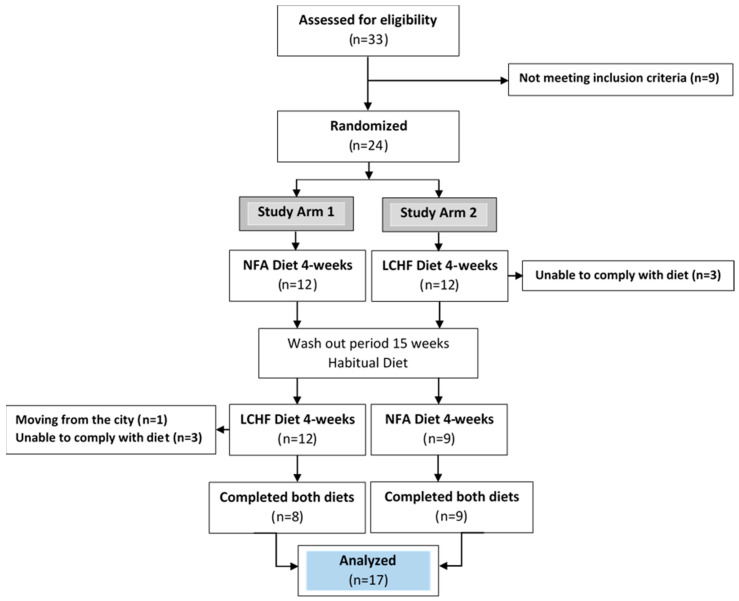
Consort flow chart. Adapted from [8,14]. LCHF diet: a ketogenic low-carbohydrate high-fat diet. NFA diet: a National Food Agency-recommended control diet.

Young, healthy, normal-weight, women consumed a ketogenic LCHF diet and an NFA-recommended control diet in random order for four weeks each, with a washout period of 15 weeks (Figure 1). The present study was originally designed to detect an effect on the primary outcome of change in LDL cholesterol. While previous crossover trials with similar outcomes have often used washout periods of 0–4 weeks [9,10,11,12], the length of the washout period in the present study was set to 15 weeks to reduce the risk of carry-over effects biasing the result. During the washout period, the participants followed their habitual diet. The primary outcome (LDL cholesterol) of this feeding study with a crossover design has been published previously [8], as well as the study protocol [13]. The outcome of the current study is body composition (secondary outcome), which was measured by dual-energy X-ray absorptiometry (DXA). This study was registered at http://www.clinicaltrials.gov/ (accessed on 10 April 2015) as NCT02417350.

### 2.2. Ethics

All participants provided written informed consent. The study protocol fulfilled the principles of the Helsinki Declaration and was approved by the Regional Ethical Review Board at Umeå University, Umeå, Sweden (Dnr 2014-361-31M and Dnr 2015-45-32M). Throughout this study, all participants had access to a research nurse to whom they could report any potential health problems. Additionally, all food provided to the participants was free of charge.

### 2.3. Eligibility Criteria, Participants, and Study Setting

We recruited healthy, normal-weight, female dietetics students aged 18–30 at Umeå University, Umeå, Sweden. A complete list of inclusion and exclusion criteria is available elsewhere [14]. The enrolment of participants was performed by a research nurse.

A total of 130 women were invited to participate, and 33 were interested in participating. Twenty-four women remained after screening and were randomized to one of two possible study arms (Figure 1) [8]. Due to seven dropouts, 17 women completed the full study. The details behind the dropouts can be found elsewhere [8]. The background characteristics of the 17 women who completed this study have been previously published and can be found in Table 1 [8,14].

This study was conducted in Umeå (a city in northern Sweden with a population of approximately 90,000).

### 2.4. Allocation and Blinding

Eligible participants were randomly allocated in a 1:1 ratio to one of the two study arms (Figure 1). To minimize the imbalance in the factor “weekly training sessions”, minimization was used for the allocation sequence [15,16]. In the minimization procedure, the first participant was allocated at random, and the following participants in the trial were allocated based on the characteristics of the participants already enrolled in such a way that the imbalance between arms considering the factor ”weekly training sessions” was reduced. The minimization procedure was performed with freely available software, namely, MINIM [17], which is a well-recommended and described allocation method (for review, see [15]), especially useful in trials with a lower number of participants. As described previously [14], the factor “weekly training sessions” had three alternatives: (1) 0–2 times, (2) 3–5 times, or (3) 6 or more training sessions per week. The vast majority exercised 3–5 times per week. The order of the two diets in study arm 1 and study arm 2 was determined by a research nurse who was not involved in the data analysis process. As a result, the researchers remained unaware of the group assignments until after the data analysis was completed and the code key was revealed. Due to the significant differences in the diets’ compositions, particularly their fat and carbohydrate content, it was not possible to blind the study participants as the meals were visibly distinct.

### 2.5. Study Diets

Details about the food and food handling, as well as photographs of the food, have been provided in the study protocol [13] and in [14]. In brief, the participants were provided with all the food and were not allowed to drink anything except water, tea, and coffee. The food was picked up at the Department of Food and Nutrition at Umeå University, and the participants were informed by a research nurse to consume all the food provided and to eat extra pre-prepared snacks with the same macronutrient distribution if they started to lose weight. Both diets contained 19% of the daily energy intake (E%) from protein. The ketogenic LCHF diet was low in carbohydrates (4E%) and high in fat (77E%), especially saturated fat (33E%). The typical food sources were meat, fish and seafood, high-fat dairy, eggs, coconut fat, olive oil, nuts, avocado, raspberries, and aboveground vegetables such as aubergine and broccoli. The control diet was based on current dietary guidelines [18], with a lot of vegetables, fruit and berries, fish and chicken, vegetable oils, low-fat dairy, and high-fiber products. The control diet was composed of 44E% carbohydrates and 33E% fat (including 10E% saturated fat). None of the participants consumed supplements or artificial sweeteners during the diet intervention.

### 2.6. Dietary Adherence and Physical Activity

Previous publications have described the dietary intake of the participants calculated from participants’ daily notes on deviations from the allotted food, as well as ketone levels in urine and blood 1 [8,14]. Worth noting is that when eating the ketogenic LCHF diet, every participant entered ketosis after a maximum of four days and that every single woman had a blood β-hydroxybutyrate concentration >0.5 mM at the end of the ketogenic diet intervention. Moreover, the physical activity levels did not differ significantly during the diet periods [14]. The measures taken to increase adherence to the diets and maintain similar levels of physical activity during the two diet periods therefore seemed appropriate. Despite these measures, both diet interventions induced significant weight loss (~3 kg and ~1 kg for the ketogenic LCHF and the control diet, respectively) [14].

### 2.7. Body Composition

Body composition was measured with DXA (Lunar iDXA, GE Healthcare, Madison, WI, USA) at the Sports Medicine Unit from day -5 to -2 (pre-diet) and day 25–27 (post-diet). The DXA was calibrated daily with a calibration block and weekly using a spine phantom, according to the manufacturer’s instructions. On arrival at the Sports Medicine Unit (approximately one hour after lunch), the participants were asked to void their bladder. For all measurements, the participants wore light clothing (underwear or shorts and sports bra), with all metal and plastic artifacts removed. The scans were conducted using the Lunar iDXA-recommended hands mid-prone protocol. The women were positioned central on the scanning bed, with arms at each side, slightly separated from the trunk, and the palm of the hand placed on the bed with their feet separated by 15 cm and supported with a Velcro strap. All measurements were taken by a single experienced and qualified technician. 

The lean mass index (LMI) was calculated by dividing the total lean mass (in kilograms) by the square of height (in meters). The appendicular lean mass index (ALMI) was calculated by dividing the appendicular lean mass (in kilograms) by the square of height (in meters). The fat mass index (FMI) was calculated by dividing the total fat mass (in kilograms) by the square of height (in meters). 

### 2.8. Statistical Analyses and Power Calculations

Power and sample size estimations for the primary outcome (LDL cholesterol) of this feeding trial have been described in the study protocol [13]. A total of 16 women were needed to complete this study, and considering a dropout rate of up to one-third, we needed to recruit 24 women. With 17 women completing this study, the goal was achieved.

The effect of the LCHF diet was estimated using a mixed effects model, with post-diet measurements of the outcome variables as the dependent variable. The fixed effects were diet and the adjustment of the pre-diet measurement. A subject-specific random effect was included to account for the dependency in outcome due to the crossover design. Further, subject-averaged pre-diet measurements were included as fixed covariates to avoid cross-level bias [19]. The analyses were performed using R version 4.3.1 (R Foundation for Statistical Computing, Vienna, Austria). The mixed models were fitted using the R function *lme* from the *nlme* package. The plausibility of normal distribution assumptions for residuals and subject-specific intercepts was assessed from the inspection of qq-plots and histograms. The assumption of the constant variance of residuals was examined by plotting them against fitted values. A separate model examining the impact of carry-over effects was fitted, where the order of intervention was included as a fixed effect.

## 3. Results

### 3.1. Dual-Energy X-ray Absorptiometry Parameters

**Figure 2:**
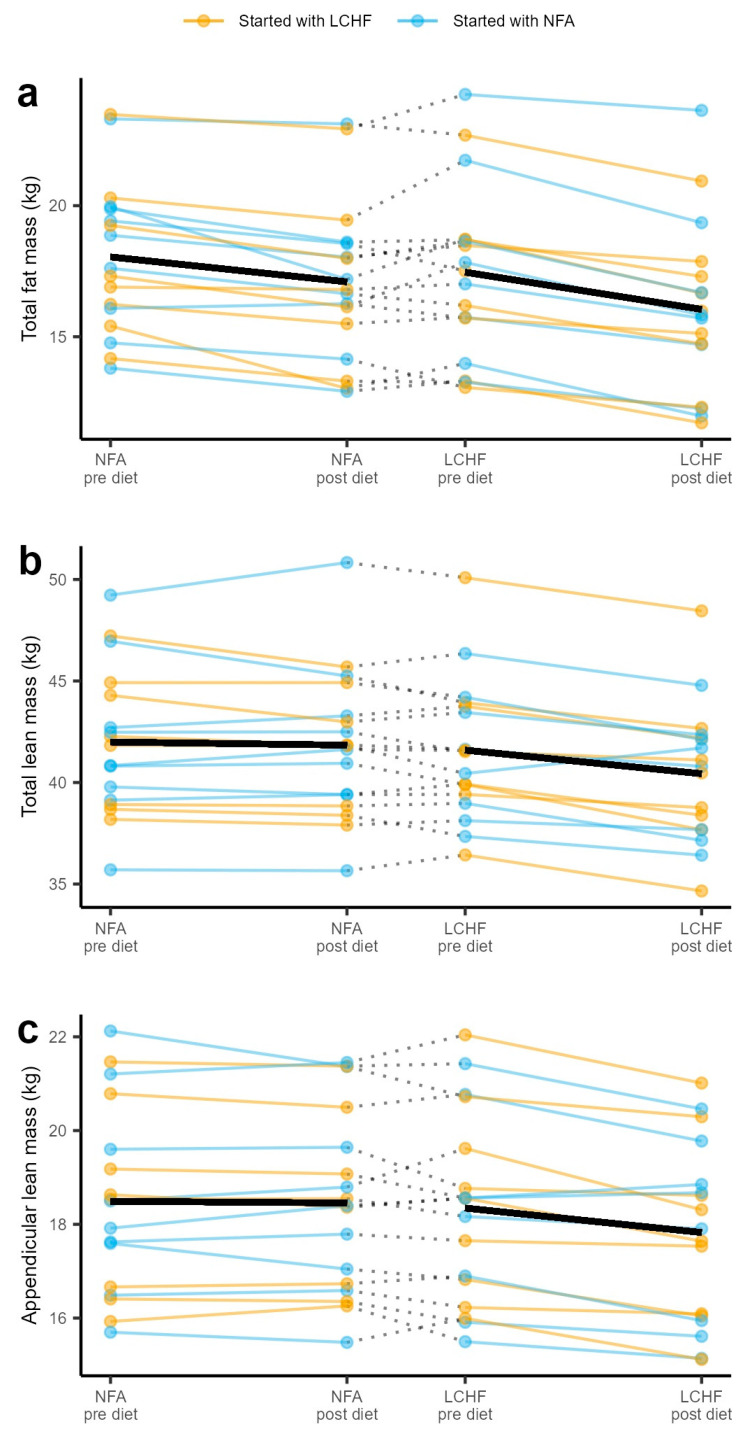
Pre- and post-diet measurements of (**a**) total fat mass; (**b**) total lean mass; and (**c**) appendicular lean mass for all individuals. The left segments show the data for the NFA diet, and the right panel shows the data for the LCHF diet. Black thicker lines represent the change in the mean for each of the diets. LCHF diet: a ketogenic low-carbohydrate high-fat diet. NFA diet: a National Food Agency-recommended control diet.

Figure 2 shows the group mean as well as individual data on/responses in total fat mass (TFM), total lean mass (TLM), and appendicular lean mass (ALM) measured by DXA. The between-participant variation in outcomes was prominent compared to within-participant effects, highlighting the benefit of using a crossover design. No apparent outliers, with excessive influence on the results, were identified. The consumption of a ketogenic LCHF diet induced a decrease in TFM (−0.66 kg, 95% confidence interval (CI): [−1.00, −0.32], *p* < 0.001), TLM (−1.45 kg, 95% confidence interval (CI): [−1.90, −1.00], *p* < 0.001), and ALM (−0.60 kg, 95% confidence interval (CI): [−0.78, −0.42], *p* < 0.001).

### 3.2. Dual-Energy X-ray Absorptiometry Indices

Descriptive values for the fat mass index (FMI), lean mass index (LMI), and appendicular lean mass index (ALMI) are given in Table 2.

## 4. Discussion

In this randomized controlled feeding trial with a crossover design among normal-weight, young, healthy women, we demonstrate that four weeks of a truly ketogenic LCHF diet reduces the lean mass twice as much as fat mass in absolute terms. Our overall conclusion is that normal-weight women adhering to a ketogenic LCHF diet should consider engaging in deliberate strength training to minimize the risk of the undesired loss of skeletal muscle.

The ketogenic LCHF diet has been proposed as an effective dietary strategy for improving body composition. Advocates of this diet emphasize that it leads to a reduction in body fat while preserving muscle mass. These effects are attributed to the metabolic shift that occurs when the body no longer has access to dietary carbohydrates. In the absence of glucose, glucagon, epinephrine, and cortisol enhance lipolysis, leading to the loss of fat mass [20]. In addition, there will be a reduction in fat mass due to the reduced fasting levels of insulin, an anti-lipolytic hormone. Furthermore, this hormonal profile, characterized by a high ratio of glucagon to insulin, will favor not only lipolysis but also gluconeogenesis and ketogenesis. The mobilization of free fatty acids from adipose tissue provides the body with fuel, not only in the form of free fatty acids but also in the form of glycerol, a gluconeogenic precursor [21]. Glycerol is one of the substrates for glucose synthesis; however, amino acids released from skeletal muscle tissue are used as substrates as well. The hormonal profile will not only activate gluconeogenetic enzymes but also augment the transfer of amino acids to the liver, which will negatively impact lean mass. Therefore, to prevent extensive loss of lean mass, it is most important that the brain, a significant consumer of glucose, switches its energy source from glucose to ketone bodies produced through increased fat oxidation [22]. Ketone bodies not only function as an alternative energy substrate but can also activate the enzyme AMP-activated protein kinase (AMPK), which increases the mitochondrial oxidation of fatty acids while simultaneously inhibiting the mechanistic target of rapamycin complex 1 (mTORC1) in skeletal tissue [23]. mTORC1 plays a crucial role in integrating growth and metabolism by responding to nutrient levels and growth signals [24]. The inhibition of mTORC1 aims to suppress energy-consuming protein synthesis in skeletal muscle tissue during glucose scarcity [23]. In summary, following a ketogenic LCHF diet impacts both fat and lean mass through hormonal changes and the molecular effects of elevated ketone bodies.

The literature shows a striking absence of studies where both activity patterns and dietary intake have been carefully controlled to evaluate the effect of a specific dietary approach on body composition. This applies even more to women. In a recent systematic review by Coleman and colleagues [25] on body composition changes in physically active populations consuming ketogenic diets, the authors discussed some limitations and risks of bias in the current literature. More specifically, they stressed the lack of randomization, the lack of a controlled food intake, and the use of insensitive techniques to measure body composition. In the current study, we have considered these common limitations in the study design. In addition, we also chose to utilize a crossover design with an extended washout period. The crossover design enhances statistical power by having participants serve as their own controls, and the extensive 15-week washout period reduces the risk of carry-over effects resulting from dietary interventions. The population used in the analysis of the present trial was the full analysis set, i.e., participants with measurements from two completed diets. In addition to the decrease in statistical power due to the loss of data, the dropout of seven participants increased the risk of bias when estimating the effect of the diets if the data are not missing completely at random (MCAR) [26]. However, considering the relatively small sample size and the complexity of the crossover data, with dependency between measurements on the same participants, we judged that multiplying imputing data from a model would not increase the validity of the results in this case.

Our current feeding study demonstrates that the ketogenic LCHF diet mediates a twofold reduction in lean mass (~1.4 kg) compared to fat mass (~0.7 kg). This occurred despite the following factors: (1) adherence to both dietary patterns was good (energy intake did not significantly differ between the two dietary interventions based on reported deviations in food intake) [14] and (2) physical activity patterns did not differ between the two dietary interventions [14]. There are several potential explanations for the increased loss of body mass during ketogenic diet intake, particularly during the first week of dietary transition. Although the body rapidly adapts to increased fat metabolism [27], there have been reports of a transient increase in energy expenditure (approximately 100 kcal/day) during the initial week of transitioning to a ketogenic diet [27], including elevated nitrogen losses and increased gluconeogenesis. Transitioning to a ketogenic diet also reduces the storage of both water and fuel in the form of glycogen [28,29,30,31]. When carbohydrate intake is minimized, muscle glycogen decreases, and consequently, the water bound to glycogen (water binds to glycogen in a ratio of approximately 3:1 [32]) is reflected as a reduced lean mass in DXA measurements. One way to minimize and account for this effect during DXA measurements would have been to introduce some form of ‘carbohydrate refeed’ before the DXA assessment [33]. However, since the intervention continued for a few more days after the DXA measurement, we chose not to compromise the nutritional ketosis with a ‘carbohydrate refeed’. An intriguing question arises: how much of the decrease in lean mass constitutes muscle mass, and how much is attributed to glycogen and glycogen-bound water? This inquiry has garnered significant interest within the field of ketogenic diets and exercise performance, particularly in studies involving men. Interestingly, the duration of adherence to a ketogenic diet appears to play a role. In a study of keto-adapted ultra-endurance runners (with an average of 20 months on the diet), Volek et al. [34] reported no significant differences in resting muscle glycogen content. Another study examined endurance-trained male cyclists who followed an LCHF diet for an extended period (>8 months), revealing a glycogen content of approximately 55–60% compared to the control group (mixed diet) [35]. Additionally, in a study where endurance-trained men followed an LCHF diet for the same duration as the women in our study (28 days), these men retained approximately 45% of muscle glycogen [28]. These measurements were conducted after an overnight fast. Assuming that the women in our study would lose 55% of total muscle glycogen (total muscle glycogen in the body is approximately 400 g), similar to Phinney and colleagues’ research [28], the actual loss of glycogen, along with glycogen-bound water, would constitute a significant portion (~600 g) of the reduction in lean mass among our female participants. Given the likelihood that the LCHF diet reduced glycogen content and, thus, total water content in the current investigation, caution is warranted when interpreting the results on lean mass. Incorporating measurements of urine osmolality using an osmometer and estimating total body water through bioelectrical impedance analysis (BIA) can provide additional insights to evaluate changes in body composition.

Even when considering the reduction in muscle glycogen, we observed an extensive decline in lean mass among the normal-weight, young women despite a high protein intake (approximately 1.8 g/kg bw/d). This decline in lean mass can even be considered surprisingly high, especially in physically active individuals and given that weight loss studies have shown that maintaining nitrogen balance can minimize the loss of lean mass [36]. In studies (predominantly involving men) with diet- and exercise-induced negative energy balance lasting up to four weeks, a protein intake of ≥1.6 g/kg bw/d supports greater retention of lean body mass [36,37,38]. However, despite our current feeding study design and the fact that reported energy intake and activity patterns did not differ significantly between the two diets, we still observed a significant decrease in both lean mass and fat mass, further emphasizing that ketogenic diets effectively reduce body weight in those who adhere to this dietary regimen. This is in line with a recent systematic review by Murphy et al. on ketogenic diets and performance in non-obese, healthy individuals (mainly men) with objectively defined ketosis, which reported a decline in body mass, fat mass, and fat-free mass [7].

In recent years, ketogenic diets have become more popular, especially among normal-weight adults and individuals pursuing aesthetic goals, including those engaged in resistance training [2]. Evidence suggests that within untrained populations, ketogenic diets may lead to a marginally higher loss of fat-free mass (FFM) compared to non-ketogenic dietary patterns [39,40,41]. Interestingly, a recent systematic review and meta-analysis examining the impact of resistance training combined with ketogenic diets on body composition also found a significant reduction in FFM among those assigned to a ketogenic diet compared to non-ketogenic counterparts [42]. The authors suggested that ketogenic diets may inhibit the mechanistic target of the rapamycin (mTOR) signaling pathway, possibly through heightened AMPK activity. Elevated AMPK activation has been shown to impede mTOR signaling, a pivotal regulator of muscle mass gains [43]. In essence, it appears that a ketogenic diet impedes anabolic pathways, although concurrent resistance exercise may help mitigate the loss of FFM. Consequently, the potential reduction in FFM remains a notable concern associated with ketogenic diets, even in the presence of concurrent resistance training [42]. Importantly, the risks associated with the loss of lean mass, primarily muscle mass, along with fat mass in female athletes, include decreased muscle strength and performance, increased risk of hormonal imbalance, menstrual irregularities, compromised immune function, sports injuries, decreased bone density, stress fractures, and long-term osteoporosis [44]. Therefore, female athletes should carefully consider these risks if adhering to a ketogenic LCHF diet.

In the most recent Position Stand on diets and body composition [3], the ISSN tried to provide clarity on the effects of various diets on body composition. The ISSN stresses that every method used to assess body composition comes with its own set of strengths and limitations. The DXA method used in the present investigation has numerous inherent strengths [3]. First, it operates as a non-invasive and expeditious technique, devoid of active subject performance, thereby streamlining the assessment process. Notably, its commendable precision and reproducibility across diverse age brackets underscore its reliability. Moreover, the method’s efficacy remains unhampered by the presence of pathological conditions or developmental anomalies, ensuring a robust and uninfluenced measurement output. In relation to the research question in the current study, the downside of DXA may be a potential limitation pertaining to its reliability in longitudinal studies involving subjects experiencing substantial fluctuations in glycogen or hydration levels between measurements. This limitation is relevant in relation to the present study, both in light of the preceding discussion regarding the effects of the ketogenic diet on muscle glycogen stores and when considering that the DXA measurements were scheduled one hour after lunch during the final week of the dietary intervention. Based on the study’s design (feeding trial), food and fluid intake in the hours before measurement was not standardized, which should be kept in mind when interpreting the results. The duration of this feeding trial was four weeks, which might be too short for the body to fully adapt to the ketogenic metabolic state. Additionally, our participants were young, normal-weight females who exercised weekly and were highly motivated to follow a strict ketogenic LCHF diet. To deepen our understanding of how this dietary regime affects body composition, future studies are warranted to investigate its long-term effects, as well as the interplay between age and lifestyle factors.

## 5. Conclusions

This unique feeding trial emphasizes the effectiveness of a strict ketogenic diet in inducing weight loss even in normal-weight healthy young women with an unaltered physical activity score. Notably, the decline in lean mass was twice as much as fat mass in absolute terms. To minimize the risk of undesired muscle mass reduction, women who adhere to a ketogenic LCHF diet should consider engaging in deliberate resistance training.

## Figures and Tables

**Table 1:** Background characteristics of the women ^1,2^.

Parameters	Median	(Min–Max)
Age	23.8	(19.7–27.3)
Body weight (kg)	60.8	(50.8–70.9)
Body height (cm)	167.6	(158.0–178.2)
BMI (kg/m^2^)	22.0	(19.4–24.0)
Waist (cm)	71.0	(67.0–78.0)
Hip (cm)	97.0	(87.5–100.0)
WHR	0.76	(0.70–0.81)
VO_2max_ (mL/kg/min)	43.65	(37.7–50.7)

^1^ This table was adapted from [8,14]. ^2^ Nine women used contraceptive hormones. BMI: body mass index; WHR: waist/hip ratio; and VO_2max_: maximal oxygen uptake. *n* = 17.

**Table 2:** Body composition indices before and at the end of the two feeding trials.

Parameters	LCHF Diet		NFA Diet	
	(Mean ± SD)		(Mean ± SD)	
	Pre-Diet	Post-Diet	Pre-Diet	Post-Diet
FMI	6.2 ± 1.1	5.7 ± 1.0	6.4 ± 0.9	6.1 ± 1.0
LMI	14.8 ± 0.8	14.4 ± 0.7	15.0 ± 0.8	14.9 ± 0.8
ALMI	6.5 ± 0.5	6.3 ± 0.5	6.6 ± 0.5	6.6 ± 0.5

Pre-diet (day -5 to -2) and post-diet (day 25–27) of the feeding trials. LCHF diet: a ketogenic low-carbohydrate high-fat diet; NFA diet: a National Food Agency-recommended control diet; FMI: fat mass index; LMI: lean mass index; and ALMI: appendicular lean mass index. *n* = 17.

## Data Availability

The data presented in this study are available upon request from the corresponding author.
